# Characterization of respiratory dendritic cells from equine lung tissues

**DOI:** 10.1186/s12917-017-1240-z

**Published:** 2017-11-06

**Authors:** Yao Lee, Matti Kiupel, Gisela Soboll Hussey

**Affiliations:** Department of Pathobiology & Diagnostic Investigation, College of Veterinary Medicine, Michigan State University, 784 Wilson Rd, A13, East Lansing, MI 48824 USA

**Keywords:** Equine, Blood dendritic cells, Lung dendritic cells, Antigen-presenting cells

## Abstract

**Background:**

Dendritic cells (DCs) are professional antigen-presenting cells that have multiple subpopulations with different phenotypes and immune functions. Previous research demonstrated that DCs have strong potential for anti-viral defense in the host. However, viruses including alphaherpesvirinae have developed strategies to interfere with the function or maturation of DCs, causing immune dysfunction and avoidance of pathogen elimination. The goal of the present study was to isolate and characterize equine lung-derived DCs (L-DCs) for use in studies of respiratory viruses and compare their features with equine blood-derived DCs (B-DCs), which are currently used for these types of studies.

**Results:**

We found that L-DCs were morphologically similar to B-DCs. Overall, B-DCs demonstrated higher expression of CD86 and CD172α than L-DCs, but both cell types expressed high levels of MHC class II and CD44, as well as moderate amounts of CD163, CD204, and Bla36. In contrast, the endocytic activity of L-DCs was elevated compared to that of B-DCs. Finally, mononuclear cells isolated from lung (L-MCs), which are used as precursors for L-DCs, expressed more antigen-presenting cell-associated markers such as MHC class II and CD172α compared to their counterparts from blood.

**Conclusions:**

Our results indicate that L-DCs may be in an earlier differentiation stage compared to B-DCs. Concurrent with this observation, L-MCs possessed significantly more antigen-uptake capacity compared to their counterparts from blood. It is likely that L-DCs play an important role in antigen uptake and processing of respiratory pathogens and are major contributors to respiratory tract immunity and may be ideal tools for future in vitro or ex vivo studies.

## Background

Dendritic cells (DCs) are the most important antigen-presenting cells (APCs) in the body. They act as a surveillance system to detect foreign antigens and shape immunogenic or tolerogenic responses [1]. There are many subsets of DCs with different phenotypes derived from either conventional or lymphoid lineages. Lymphoid lineage DCs primarily differentiate into plasmacytoid DCs and occupy approximately 0.5% of peripheral blood mononuclear cells (PBMCs) in humans [2], but the cell population percentage is unclear in horses. Conventional lineage DCs generally differentiate into myeloid DCs which originally come from tissues, such as epithelial or interstitial DCs. Blood monocyte-derived DCs (B-DCs), as one group of myeloid DCs, can be generated by incubation of monocytes that are isolated from PBMCs with exogenous granulocyte macrophage colony-stimulating factor (GM-CSF) and interleukin-4 (IL-4) for 6–7 days [3]. This approach produces a highly-differentiated DC population, which is specialized in antigen presentation and T cell priming [3–5].

Studies in humans and mice have shown that conventional DCs isolated and cultured from different tissues including bone marrow, lung, gut, and other organs, possessed slightly different phenotypes compared to B-DCs [6–10]. As one example, the respiratory tract represents one of the largest surface areas in the body and acts as an interface with the external environment that is frequently exposed to foreign particles or pathogens. For immune defense, the respiratory tract contains DCs that function as a robust antigen presentation system. Human lung DCs are localized within the airway epithelium, alveolar septae, or connective tissues of the pulmonary parenchyma [7]. Lung DCs are typically isolated from either bronchoalveolar lavage fluid (BALF) or by lung tissue digestion, resulting in a number of phenotypes and sub-populations [11, 12]. Interestingly, airway derived DCs were found to possess better antigen presenting capacity than DCs isolated from the blood [7]. It has also been shown that lung DCs, which reside in the intraepithelial region, can extend their processes through the luminal surface into the airway to detect any foreign antigens [13]. More recent studies suggested that DCs derived from tissues without “danger” signal stimulation should be regarded as immature DCs, based on their major role in antigen uptake and endocytosis of antigens [11, 14]. However, at this point, the phenotype and function of DC from different sources is not well understood for many veterinary species including horses, and most studies use B-DCs for investigating veterinary diseases.

As the bridge between the innate and adaptive immunity, DCs can direct the outcome of infectious diseases such as bacteria, fungi, parasites or viruses [15–17]. However, many viruses, including herpesviruses, have strategies to interfere with DC function through the down regulation of the host immune response. Human herpes simplex virus (HSV) inhibits DC maturation by modulating the expression of co-stimulatory molecules on DC, which consequently leads to the absence of cytokine production and lack of migration back to lymphoid organs [18]. Virion host shut-off protein from the tegument of HSV-1 has been found to impair DC activation via a Toll-like receptor-independent pathway [19]. Equine herpesvirus-1 (EHV-1) is a major viral pathogen of horses and the cause of rhinopneumonitis, abortion, and central nervous system disorders. Because the respiratory epithelium is the first site of contact between host and pathogen, as well as the initial site for viral replication, it is important to understand respiratory tract immunity including the sentinel network of DCs if we are to understand immunity to EHV-1. Recent research has shown that EHV-1 interferes with the migration of monocytes and DCs isolated from the airway mucosa and uses these cells for transport from the apical side of the respiratory epithelium to the lamina propria and for establishment of viremia [20]. However, the exact mechanism of this process has yet to be identified and protocols for isolating respiratory dendritic cells at numbers sufficient for further in vitro or ex vivo use are needed, particularly in veterinary species.

Because it is likely that characteristics of blood-derived DCs will be different from those of lung-derived DCs, the objective of the current study was to culture lung-derived DCs by adapting the protocol used for isolating blood DCs and to characterize the isolated cells in addition to comparing them with blood-derived DCs. For this purpose, mononuclear cells from equine lung tissue were isolated and cultivated with equine recombinant GM-CSF and IL-4 to generate lung DCs. Isolated cells were then characterized using common markers for APCs [3, 4] and endocytosis was evaluated.

## Methods

### Animals and sample collections

Lungs from 3 adult horses, and blood from 4 different adult horses were collected for this study. None of the horses showed signs of respiratory diseases, and horses used for lung collection were euthanized for unrelated reasons. Horses were of mixed breed, both genders and ranged in age from 2 to 23 years. Euthanasia was performed by an overdose of 0.22 ml/kg of a 39 mg/ml sodium pentobarbital solution as previously described [21]. All experimental protocols were reviewed and approved under number AUF 10/15–160-00 and AUF 05/13–111-00 by the Michigan State University Institutional Animal Care and Use Committee. During the necropsy procedure, a sample of lung tissue from the diaphragmatic lobe measuring approximately 20 × 20 × 20 cm was collected from each horse. Tissues were washed with cold phosphate buffered saline (PBS) [Gibco, Carlsbad, CA, USA] and deposited in cold DMEM [Gibco, USA] for transportation. Five-hundred ml of 0.1% heparin (1000 U/ml) [Sagent Pharmaceuticals, Schaumburg, IL, USA]-anticoagulated blood was collected from each horse for PBMC isolation.

### Lung tissue processing

Freshly collected lung tissue was processed as previously described with some modifications [12, 14]. The tissue was washed with sterile cold PBS several times to remove blood. Tissue was then minced to small pieces (approximately 0.2 × 0.2 × 0.2 cm) and soaked in digestion media (1 mg/ml collagenase type 2 [Sigma-Aldrich, St. Louis, MO, USA], 0.02 mg/ml DNase I [Life technologies, Carlsbad, CA, USA], 5% fetal bovine serum (FBS) [Gibco, USA] and 1% penicillin/ streptomycin [Gibco, USA] dissolved in RPMI-1640 with L-glutamine and 2-mercaptoethanol [Gibco, USA]) for 2–4 h at 37 °C. The digestion media was replaced one time during the incubation to enhance the digestion effect. Following this incubation period, cold 10% FBS was added to the tissue suspension to inactivate the digestion media enzymes. The tissue suspension was then passed through a 100 μm sieve followed by 40 μm cell strainers [Greiner Bio-One, Monroe, NC, USA] to remove tissue debris, and centrifugation was performed on the flow-through at 300 g for 10 min at room temperature. After discarding the supernatant, the cell pellet was re-suspended in PBS and was subject to mononuclear cell isolation (see section 2.4).

### Isolation of peripheral blood mononuclear cells, monocytes, and differentiation of monocyte-derived dendritic cells from blood samples

The protocol for isolating different subsets of cells from equine whole blood has been described previously [3]. Briefly, PBMCs were isolated from heparinized whole blood by density centrifugation at 600 g for 45 min at room temperature with Histopaque (∂ = 1.077) [Sigma, USA]. Then, PBMCs were re-suspended and cultured in culture media (cRPMI) (RPMI 1640 with 4 mM L-glutamine, 10% FBS, and 1% penicillin / streptomycin [Gibco, USA]) at a concentration of 1 **×** 10^7^ cells/ml for 4 h in 200 mm tissue culture-treated dishes at 37 °C. Following the incubation period, adherent blood monocytes (B-MOS) were separated from non-adherent mononuclear cells by washing off non-adherent cells with cRPMI. To collect the adherent B-MOS for flow cytometry, the B-MOS were subjected to treatment of cold Versene EDTA solution [ThermoFisher, Rockford, IL, USA] to detach, followed by gently scraping to collect the cells. For B-DC culture, dendritic cell culture media (RPMI-DC) (RPMI 1640 with 4 mM L-glutamine, 10% heat-inactivated heterologous horse serum, 50 uM β-mercaptoethanol [Sigma, USA], 1% penicillin / streptomycin, and 2.5 μg/ml amphotericin B [Gibco, USA], supplemented with recombinant equine GM-CSF (10 ng/ml or 1000 U/ml) as well as recombinant equine IL-4 (10 ng/ml or 1000 U/ml) [KingFisher Biotech, St. Paul, MN, USA], was added and adherent B-MOS were incubated for 4 days, at which point cells differentiated into B-DCs. Loosely adherent B-DCs were separated from firmly attached cells, and purified by further density centrifugation at 600 g for 15 min with Nycoprep (∂ = 1.068) [Progen Biotechnik, Heidelburg, Germany] [4]. Low-density DCs were then collected and cultured in RPMI-DC supplemented with GM-CSF and IL-4 for 3 more days.

### Isolations of mononuclear cells, monocytes, and dendritic cells from lung tissues

Similar to the procedure applied for isolation of B-MOS and B-DCs (section 2.3), density centrifugation was performed on the cell suspension obtained from lung tissues at 600 g for 45 min with Histopaque (∂ = 1.077). Cells isolated in this manner were defined as lung mononuclear cells (L-MCs), which were then further cultured in cRPMI for 4 h to separate adherent lung monocytes (L-MOS) from non-adherent cells. For flow cytometry of adherent L-MOS, cells were subjected to treatment of cold Versene EDTA solution [ThermoFisher, USA] followed by gently scrapping for collection. For generation of L-DCs, adherent L-MOS were continuously cultured with RPMI-DC supplemented with GM-CSF and IL-4 for a total of 5 days, with a one-time replenishment of fresh media during cultivation. Cells were collected as lung-derived dendritic cells (L-DCs) after 5 days of cultivation.

### Antibodies for flow cytometric analysis and immunocytochemical (ICC) labeling

Different cell types were characterized using antibodies to cell antigens that are generally regarded as monocytic and dendritic cell markers (Table 1). Equine-specific monoclonal antibodies (mAb) recognizing equine MHC class II (clone CVS10) and CD44 mAb (clone CVS18) have previously been described [22]. An anti-CD172α mAb, produced by Washington State University (clone DG-DH59B) [Cat. No. DG-BOV2049] has shown cross-reactivity with multiple animal species [23]. Furthermore, anti-human mAbs with cross-reactivity to equine CD86 (clone IT2.2) [Cat No. 555663; BD Pharmingen, San Jose, CA, USA]) [3], CD163 (clone AM-3 K) [Cat. No. KT-013; Trans Genic, Kobe, Japan], CD204 (clone SRA-E5) [Cat. No. KT-022; Trans Genic, Japan], and anti-B Lymphocyte antigen 36 mAb (Bla 36, clone A7–42) [Cat. No. MU231-UC; Biogenex, Fremont, CA, USA] were used. Mouse IgG1 and IgG2b isotype antibodies [ThermoFisher, USA] were applied as isotype controls. A fluorescein FITC AffiniPure goat anti-mouse IgG H + L [Jackson ImmunoResearch, West Grove, PA, USA] was used as the secondary antibody for flow cytometric analysis.

**Table 1:** The summary of antigens applied to characterize various types of cells isolated from blood and lung tissues in the present study

Antigen	Function
MHC class II	Antigen presenting protein on antigen presenting cells
CD44	Cell adhesion molecule on lymphocytes, monocytes, and DCs
CD86	T cell co-stimulating molecule on DCs
CD163	Macrophage scavenger receptor on cells of monocytic lineage
CD172α	Signal-regulatory protein on myeloid cells
CD204	Macrophage scavenger receptor on cells of monocytic lineage
Bla36	Glycoprotein on B cell lineage, macrophages, and DCs
IgG1&2b	Isotype controls

### Flow cytometric analysis for detection of cell surface antigens

Flow cytometric analysis was performed to quantify expression of MHC class II, CD44, CD86, and CD172α. Cells were collected and re-suspended in FACS buffer (PBS with 0.4% bovine serum albumin and 0.1% sodium azide) [Sigma, USA] prior to incubation with the respective primary antibodies or isotype control for 1 h at 4 °C, at appropriate concentrations: mAbs for MHC class II and CD44: no dilution; CD86: 1:10 dilution; CD172α: 1:100 dilution. Isotype controls were applied at the same concentration as mAbs. After 3 washes, cells were re-suspended in 1:300 diluted goat anti-mouse IgG secondary antibody and incubated for 1 h at 4 °C, before analysis via BD Accuri™ C6 cytometer [BD Biosciences, San Jose, CA, USA]. Gating was applied in forward scatter versus side scatter (FSC/SSC) dot plots to exclude cell debris. Autofluorescence or background was excluded by examining the fluorescence of unstained cells and isotype controls.

### Endocytosis tracer assay

An endocytosis tracer assay was used to determine the antigen uptake and endocytic capacity of the different isolated cell types [24]. A total number of 4 × 10^5^ cells was suspended in 200 μl of RPMI-1640 blank or fluorescently tagged antigens (50 μg ovalbumin conjugated with Alexa Fluor 647™ [Molecular Probes, Eugene, OR, USA]) and incubated for 1.5 h at 37 °C or 4 °C, in which the latter were considered as the negative control. Cold FACS buffer was added to terminate the endocytosis reaction, followed by 3 washes in FACS buffer. Flow cytometric analysis was conducted using a BD Accuri™ C6 cytometer [BD Biosciences, San Jose, CA, USA]. For analysis of the results, both percentage of cells stained positive compared to controls and the mean fluorescence intensity (MFI) of cells incubated at 37 °C standardized against the MFI of cells incubated at 4 °C were evaluated. Results were presented as MFI of experimental samples compared to MFI of controls as previously described [24].

### Immunocytochemical (ICC) labeling

For ICC labeling, mAb for CD163, CD204, and Bla36 were used. Specific cell-types were re-suspended in PBS, and subjected to an 800 rpm, 10 min centrifugation with a Cyto-Tek^®^ Cytocentrifuge [Electron Microscopy Sciences, Hatfield, PA, USA] to generate cytospin slides, with 5 × 10^4^ cells per slide. Slides were fixed by immersion in acetone at -20 °C for 5 min. Slides were then submitted to the Michigan State University Diagnostic Center for Population and Animal Health for ICC labeling. An EnVision FLEX+ detecting system [Dako, Carpinteria, CA, USA] including peroxidase block, non-biotin polymerized horseradish peroxidase (HRP), 3,3′-Diaminobenzidine (DAB) with DAB plus chromogen solution, and FLEX+ mouse linker, were applied with an autostainer [Dako, USA] as previously described [25]. Hematoxylin [Dako, USA] was used as the counter stain. Finally, cytological inspection was performed on a light microscope [Leica, Buffalo Grove, IL, USA] and evaluated by a board certified pathologist. The general microscopic observation was performed under 100× magnification to inclusively evaluate the positive staining of the whole cell population. A grading system was used to determine the positive percentage of the detected surface antigens, as follows: one plus for less than 30% positive cells, double plus for 30% to 60% positive cells, triple plus for more than 60% cells that were positive, minus for no positive cells (negative result).

### Statistical analysis

Data was graphed using Excel 2016 [Microsoft, Redmond, WA, USA]. For the endocytosis assay, one-way ANOVA followed by post-hoc Tukey’s multiple comparisons test [Graphpad Prism 6.01 for Windows, La Jolla, CA, USA] was used to compare the values of mean fluorescence intensity (MFI) in a log scale among different cell types. Statistical significance was considered at *p* < 0.05.

## Results

### Morphology of mononuclear cells and dendritic cells

**Fig. 1:**
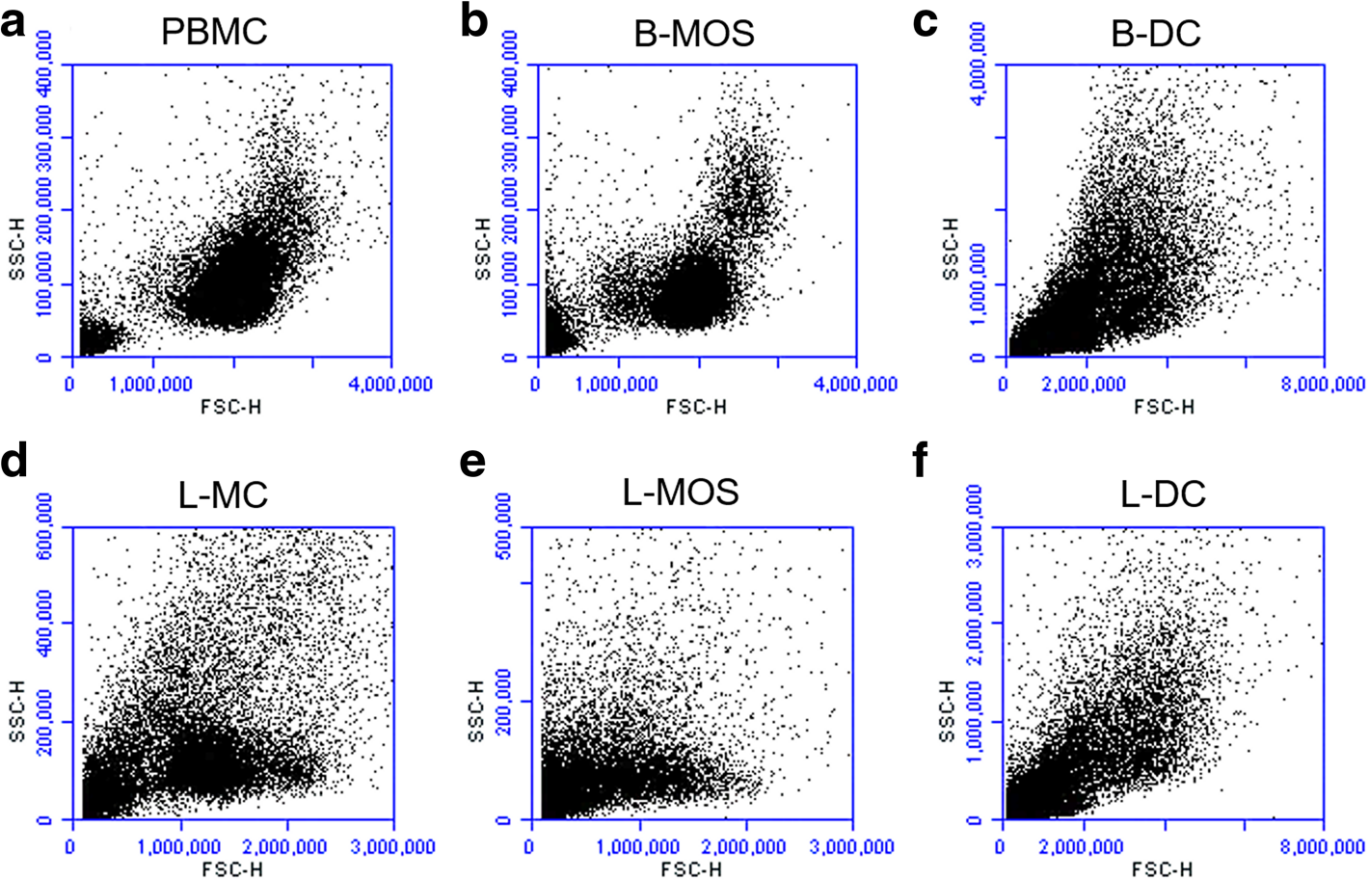
The dot plots of forward scatter-height (FSC-H) versus side scatter-height (SSC-H) from the representative sample, respectively, for all the cell types isolated in this study (**a**. PBMCs, **b**. B-MOS, **c**. B-DC, **d**. L-MC, **e**. L-MOS, **f**. L-DC

**Fig. 2:**
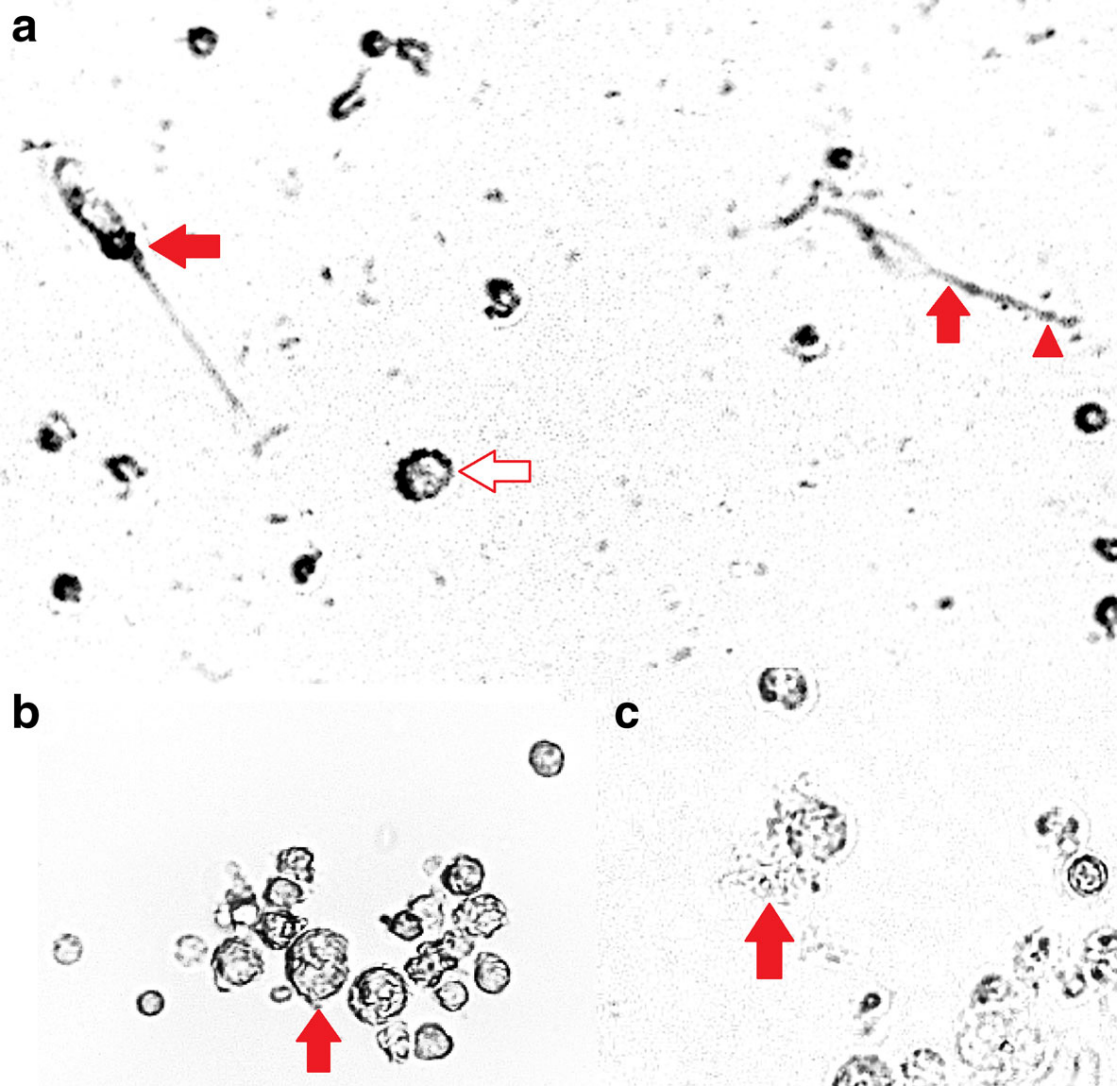
Microscopic images of dendritic cells following cultivation with recombinant granulocyte macrophage colony-stimulating factor (GM-CSF) and recombinant interleukin-4 (IL-4). All of the images were taken at 200× magnification. **a**) Blood dendritic cells (B-DCs) collected after 3 days of incubation with recombinant proteins of GM-CSF and IL-4. Solid arrows show cells with extended dendrites, one of which possessed several nodes aligning (indicated by arrowhead). Some cells had short projections on the surface (indicated by hollow arrow). **b**) B-DCs collected after 7 days of incubation with recombinant GM-CSF and IL-4. The cells were veiled with pseudopods (indicated by solid arrow). **c**) Lung dendritic cells (L-DCs) after 5 days of incubation with GM-CSF and IL-4. Cells demonstrated visible pseudopods (indicated by solid arrow)

Microscopically, freshly isolated PBMCs were round-shaped with a moderate amount of cytoplasm and an euchromatic nucleus (data not shown). Approximately 5–10% of PBMCs, which were considered B-MOS, adhered to the bottom of the tissue culture plate after 2–4 h incubation at 37 °C while the remaining cells were non-adherent were removed after 4 h of incubation. The B-MOS had similar morphology when compared to PBMCs in suspension, but were slightly larger in cell size (data not shown). Upon evaluation by flow cytometry in FSC/SCC plots, PBMCs consisted of mainly lymphocytes characterized by small size (FSC^low^) and low granularity (SSC^low^), and a small portion of monocytes characterized by larger size (FSC^moderate^) and higher granularity (SSC^moderate^) (Fig. 1a). After 4 h attachment, an increased number of monocytes, which were FSC^moderate^ and SSC^moderate^ was observed (Fig. 1b),

Initially there were no visible projections on the cell surface of B-MOS. However, projections, or pseudopods, became visible after several days in the presence of GM-CSF and IL-4, suggesting that the cells were transforming into DCs. Dendritic cells were polymorphic during the culture period. They possessed numerous short projections on the cell surface (Fig. 2a, hollow arrow), sometimes with single or multiple extremely extended dendrites (Fig. 2a, solid arrows) aligned by several nodes (Fig. 2a, arrow head). The cells became loosely attached or floated by day 4, and could be easily separated from firmly attached remaining cells. By the end of 7 days in culture, B-DCs were identified as non-adherent, polymorphic and veiled in shape, with several pseudopods projecting around the cell surface (Fig. 2b, solid arrow). Upon evaluation by flow cytometry in FSC/SCC plots, live B-DCs were mostly large in cell size (FSC^high^) and moderate to high in granularity (SSC^high^) compared to lymphocytes and monocytes (Fig. 1c).

The morphology of cells isolated from the lungs was comparable with those from blood samples. L-MCs isolated by Histopaque [Sigma, USA] centrifugation were small, round, non-adherent cells, with scarce to moderate cytoplasm and medium size nuclei. The flow cytometry results showed a main cell population characterized by small size (FSC^low^) and low granularity (SSC^low^), and a small group of cells characterized by slightly larger size (FSC^moderate^) and low granularity (SSC^low^) (Fig. 1d). After a 2–4 h incubation at 37 °C, adherent L-MOS had identical morphology when compared to B-MOS. Similar to L-MCs, flow cytometry for L-MOS revealed a main population characterized by small size (FSC^low^) and low granularity (SSC^low^), and few cells exhibiting larger size (FSC^moderate^) and low granularity (SSC^low^) (Fig. 1e).

L-DCs did not exhibit pseudopods until cultivation with GM-CSF and IL-4 for several days. Like B-DCs, L-DCs were loosely attached or floating, polymorphic, with either remarkably extended or short projections around cell surface (Fig. 2c, solid arrow). Upon evaluation by flow cytometry using FSC/SCC plots, the live cell population of L-DCs was characterized by large cell size (FSC^high^) and moderate to high granularity (SSC^high^) (Fig. 1f).

### Immunophenotypes of mononuclear cells, monocytes, and dendritic cells isolated from blood and lungs

The immunophenotypes of the different isolated cell types were examined by flow cytometry and immunocytochemical (ICC) labeling. The average background staining for flow cytometry assays ranged from 3 to 10% depending on cell type. Results with values below 10% were considered negative.

**Fig. 3:**
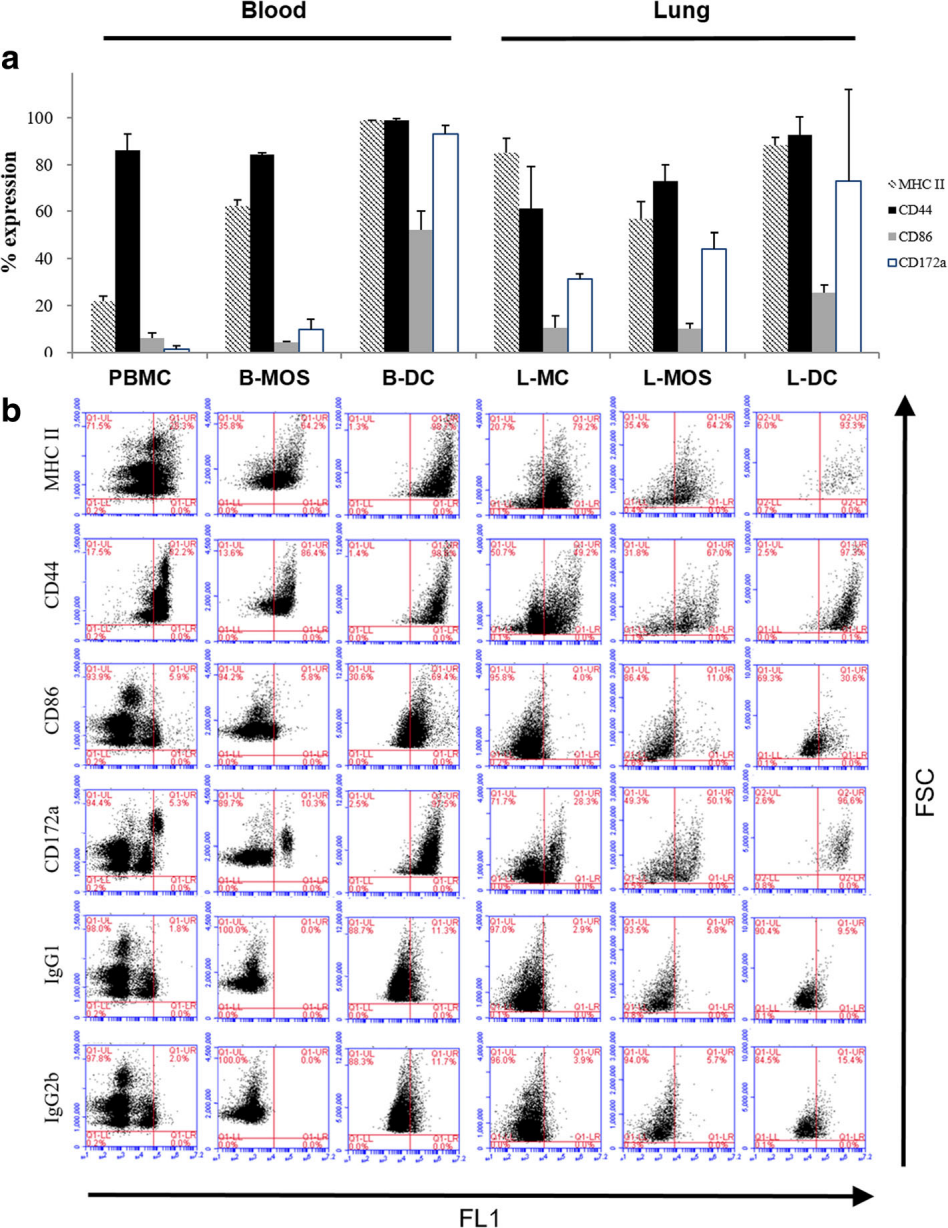
Results of flow cytometric analysis. **a**. Bar chart showing mean values of immunofluorescence of MHC class II (box filled with diagonal pattern), CD44 (solid black box), CD86 (solid gray box), and CD172α (white box) expression in peripheral blood mononuclear cell (PBMCs), blood monocyte (B-MOS), and blood dendritic cell (B-DC), as well as lung mononuclear cell (L-MC), lung monocyte (L-MOS), and lung dendritic cell (L-DC). The error bars indicate standard deviation. **b**. Representative results showing dot plots of blood cells (PBMC, B-MOS, and B-DC) from one horse and lung cells (L-MC, L-MOS, and L-DC) from another horse. The X-axis for each dot plot is a logarithmic scale of immunofluorescence parameter (FL1) for MHC class II, CD44, CD86, CD172a, and isotype controls including IgG1 and IgG2b; the y-axis for each dot plot is forward scatter (FSC), indicating the size of the cells

A summary of the results of the flow cytometric analysis is shown in Fig. 3, with percentage of cells expressing each cell marker for each cell group shown as a bar chart (Fig. 3a). Mean fluorescence intensity (MFI) generally correlated with percentage values. Figure 3b shows representative dot plots of FSC (cell size) versus immunofluorescence (FL1) of each cell marker for each cell group isolated from blood and lung cells, respectively. The dot plots highlight a trend for cells becoming more uniform in phenotype following the cell adherence step and cultivation with GM-CSF and IL-4 (Fig. 2b B-DCs and L-DCs).

**Fig. 4:**
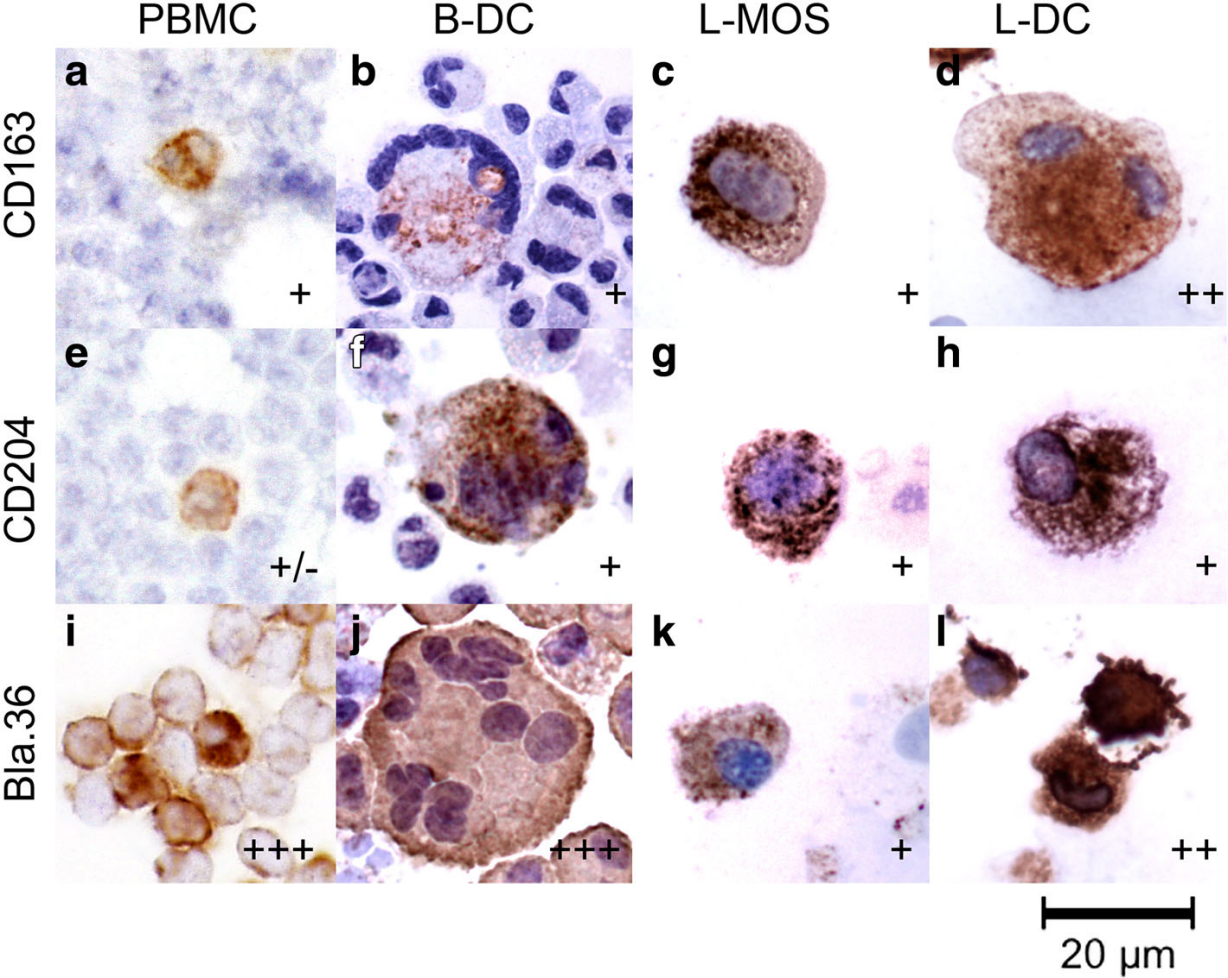
Microscopic images of immunocytochemical staining for CD163 (**a**-**d**), CD204 (**e**-**h**), and Bla36 (**i**-**l**) expression on blood monocytes (B-MOS) and blood dendritic cells (B-DCs), as well as lung monocytes (L-MOS) and lung dendritic cells (L-DCs). All of the images were taken at 400× magnification

Furthermore, we show that PBMCs had low expression of MHC class II (21.8%), high expression of CD44 (86.0%), and no expression of CD86 and CD172α (Fig. 3a). In contrast, expression of MHC class II and CD172a was elevated in B-MOS while CD44 and CD86 stayed the same. Immunocytochemically, PBMCs expressed similar cell surface molecules when compared to B-MOS, which were positive for Bla36^+^ but only mildly positive for macrophage scavenger receptors e.g. CD163 and CD204 (Fig. 4-a, −e, −i). B-DCs strongly expressed MHC class II (98%), CD44 (99%), and CD172α (93%), as well as moderately expressed CD86 (52.3%) (Fig. 3a). B-DCs were also Bla36^+++^, and some of the giant cells with multiple nuclei were positive for CD163 and CD204 (Fig. 4-b, −f, −j).

For cells isolated from the lung, a moderate to high percentage of L-MCs expressed MHC class II (84.8%), CD44 (61.3%), and CD172α (31.5%), but were negative for CD86 using flow cytometric analysis (Fig. 3). Immunocytochemically, L-MCs also showed moderate expression of CD163^+^, CD204^+^, and Bla36^+^ (data not shown). Results of L-MOS were similar to L-MCs; L-MOS expressed MHC class II (56.8%), CD44 (72.9%), and CD172α (44.1%), but were negative for CD86 (Fig. 2), as well as CD163^+^, CD204^+^, and Bla36^+^ (Fig. 4-c, −g, −k). Expression of most markers was increased on L-DCs, which showed a high percentage of cells expressing MHC class II (88.3%), CD44 (92.7%), CD172α (73.1%), and moderate percentage of cells expressing CD86 (25.3%) using flow cytometric analysis (Fig. 3). L-DCs were also positive for CD163^++^, CD204^+^, and Bla36^++^ (Fig. 4-d, −h, −l).

### Antigen uptake by dendritic cells isolated from lung and blood

**Fig. 5:**
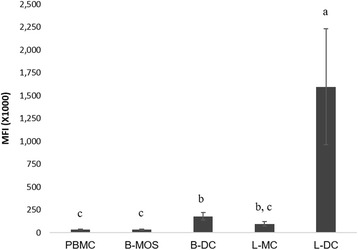
Flow cytometric analysis showing antigen uptake capacity of blood mononuclear cell (PBMC), blood monocyte (B-MOS), blood dendritic cell (B-DC), as well as lung mononuclear cell (L-MC) and lung dendritic cell (L-DC). Mean values are shown for each cell type with error bars indicating standard errors of the mean (SEM). Different alphabetic letters represent significant differences (*p* < 0.05) using Tukey’s multiple comparison tests. MFI: mean fluorescence intensit

For endocytosis assays, PBMCs and B-MOS were isolated from 4 horses, and B-DCs were isolated from 3 horses. L-MCs and L-DCs were isolated from 3 additional horses. Percentage of cells that stained positive and MFIs were compared among different cell types and trends between percentage of positive cells and MFI were similar (data not shown). L-DCs demonstrated significantly higher antigen uptake when compared to any other cell type (*p* < 0.05). The antigen uptake of B-DCs was significantly higher than PBMCs or B-MOS (*p* < 0.05) but there was no significant difference compared to L-MCs (*p* = 0.5895) (Fig. 5). An endocytosis assay was not performed on L-MOS because of limited cell numbers.

## Discussion

The majority of studies that investigate viral interactions with host DCs have used cells derived from blood leukocytes because of the relative ease of obtaining appropriate cell numbers and generating cells with DC phenotype [3, 4]. However, DCs derived from respiratory tissues may have distinct immunological phenotypes as well as better capacity for antigen processing when compared to blood-derived cells [26, 27]. Thus, it may be advantageous to use lung-derived DCs particularly when studying the interaction between pathogens and antigen-presenting cells that infect via mucosal routes, or when considering pathogens that can affect DC function [18–20]. We developed a protocol for isolating L-DCs from lung mononuclear cells by adapting a commonly used equine B-DC protocol [3]. While the L-DC derived using this protocol maybe distinct from tissue resident lung DCs isolated directly from tissue lysates [7, 11, 14, 28], the number of tissue resident lung DCs isolated from lysates is often scarce and cells die quickly without supplement of GM-CSF [29]. Our protocol is the first to generate cytokine-activated lung DCs from horses at sufficient numbers that are phenotypically similar to B-DCs and have improved endocytic capacity.

More specifically, we isolated and cultivated L-DCs by using a protocol similar to isolation of B-DC with small modifications. Comparing DCs derived from blood and lung, we demonstrated that the morphology of DCs was similar; however, the immunological profiles of L-DCs were different compared to B-DCs. L-DCs expressed high levels of MHC class II and CD44 that were similar compared to the expression profiles of B-DCs, though the expression level of CD86 was higher on B-DCs (52.3%) than on L-DCs (25.3%). MHC class II is responsible for antigen presentation and a marker found in many cell types; high levels of this marker are indicative of antigen presenting cells, in particular DCs. CD44 is a cell surface glycoprotein involved in lymphocyte/monocyte activation, cell-to-cell interaction and migration. When there is antigen present, the expression of CD44 is triggered to bind extracellular matrix, inducing an inflammatory cell response [30]. The expression of CD44 on DCs can also be enhanced by antigen stimulation, which results in DC migration and mediates T cell activation [31]. CD86 facilitates DC stimulation and contact with the T cell receptor [32]. Peripheral DCs are typically immature DCs, generally in charge of antigen uptake through receptor-mediated endocytosis and function in the transport from sites of infection to lymph nodes. Once they arrive in the lymph nodes, DCs present processed antigen to T cells via MHC class II molecules and provide co-stimulation signals via CD86 and T cell receptor CD28 [33]. L-DCs isolated in our study expressed less CD86 than B-DCs did, suggesting that L-DCs may be less mature when compared to B-DCs. We also observed that initially the isolated mononuclear cells (B-MOS or L-MOs) were of a mixed cell type; however, the phenotype of cells became more uniform after cultivation with GM-CSF and IL-4, suggesting a role of these cytokines in the maturation of B-DCs and L-DCs (Fig. 3b).

CD172α belongs to a family of signal regulatory proteins and is primarily expressed on myeloid cells including monocytes, DCs, and macrophages, though the expression is different among different DC subtypes or in different tissues [34–36]. A recent equine study further illustrated that APCs isolated from airway epithelium had both CD172α^+^ and CD172α^−^ cells [26]. L-DCs in our study expressed moderate to high level of CD172α, but with remarkable variability when compared to B-DCs. CD172α is involved in DC migration and activation of Th2 cells [37] and can be used as an indicator of differentiation and maturation of DCs. In general, our results suggest that L-DCs showed similar expressions of markers that were expressed on B-DCs, but at lower levels. This could be due to there being multiple cell sub-types within each cell population or due to a lesser degree of maturation of L-DCs when compared to B-DCs. More experiments using dual or triple staining of classical DC markers such as CD11c, CD86, CD172a and CD14 could be performed to further subtype these populations but this went beyond the scope of the current study [5].

L-MCs and L-MOS shared similar marker profiles, with moderate to high level of MHC class II and CD44 expressions, moderate levels of CD172α, and low expression of CD86, indicating that mononuclear cells and monocytes isolated from lung tissues express the molecules associated with antigen presentation and DC maturation and that there is already a number of L-MOS present in the isolated L-MCs. In contrast and as expected, PBMCs demonstrated a distinct profile with high expression of CD44, but low to no expression of DC markers such as MHC class II, CD86, and CD172α. It was also noticed that mononuclear cells isolated from lung tissues possessed more features consistent with antigen presentation than their counterparts isolated from blood prior to stimulation with cytokines. Studies in either human or animal species have demonstrated isolation of different DC subpopulations directly from lung tissue digests without further cultivation [12, 14, 24, 27], verifying that naïve APCs are widely distributed in tissues, but less so in blood.

Cytokine supplementation during DC cultivation and differentiation is critical to produce differentiated cell populations for ex vivo cell culture. Continuous replenishment of GM-CSF and IL-4 in culture was found essential to keep high expression of MHC I and II on DCs, as well as to increase the level of antigen uptake via receptor-mediated endocytosis [38]. In our study, equine DCs isolated from digested lung tissues cultured with GM-CSF and IL-4 showed higher capacity of endocytosis than those without GM-CSF and IL-4 supplementation. Previous studies have demonstrated that the endocytic abilities of CD172α^+^ APCs and CD172α^−^ APCs were not different from each other when no cytokines were added to cell culture [26]. It is likely that immature DCs prior to stimulation by danger signals such as CpG or LPS [39] have good endocytic ability to internalize antigens. After migrating to lymph nodes, they require inductive signals, including GM-CSF, TNFα, and CD40L, for further maturation to full antigen presenting capacity and activation of T cells [28]. Our results suggest that GM-CSF and IL-4 play a role in inducing coordination of DC maturation. Moreover, the endocytosis level of L-DCs was higher than that of B-DCs. The results of the endocytosis assay, in parallel with the results of phenotyping, suggest that B-DCs are possible more mature/differentiated than L-DCs, though both L-DCs and B-DCs are still considered relatively immature DCs due to lack of danger signal induction [11]. It is however important to consider incubation periods with GM-CSF and IL-4 for L-DCs were only 5 days rather than 7 days for the B-DCs, due to poor cell viability of L-DCs after more than 5-days of culture. This may explain the lesser degree of maturation/differentiation of L-DC. Previous studies demonstrated that a continuous supplement of GM-CSF and IL-4 for 6–7 days is required to generate stable phenotype of DCs [40]. Therefore, different phenotypes of B-DCs and L-DCs may be attributed to discrepancy of duration of cytokine stimulation, aside from differences in cell origin.

CD163 and CD204 are members of a super family of scavenger receptors; CD163 belongs to class B, whereas CD204 belongs to class A (also known as SRA). Both CD163 and CD204 are considered to be specific markers for tissue-resident macrophages [41, 42], particularly anti-inflammatory macrophages M2 [43, 44]. More recent studies have shown expression of CD163 and CD204 on ex vivo generated DCs [44–46]. In our study, cells exhibiting APC phenotypes, including B-DCs, L-MOS, and L-DCs, stained positive for CD163 and CD204. Within these cell types a small portion of cells, which showed multinucleated giant cell morphology expressed strong signals for CD163 and CD204. Considering that M2 macrophages can be generated from PBMCs by stimulation with macrophage colony stimulating factor (M-CSF), IL-4, and IL-10 [47], which are similar to the cytokines used for culture of DCs, it might be possible that our isolated cell populations contained a portion of M2-associated macrophages. BLA36, on the other hand, is a surface glycoprotein that has been reported primarily on activated B lymphocytes, Reed–Sternberg cells and their mononuclear variants isolated from human Hodgkin’s lymphoma [48], as well as DCs in dog skin [49]. Our present study shows that a portion of equine mononuclear cells and DCs from equine tissues were BLA36 positive, which has been shown to also label macrophages and dendritic cells in horses [50]. Further cell sorting plus characterization will be required to identify sub-populations of L-DCs.

## Conclusions

The phenotypes and functions of lung-derived DCs isolated in our study will be particularly helpful for future research of respiratory diseases. Our study used an already established protocol for isolating B-DCs by culture with GM-CSF and IL-4 and generated a population of cells from the horse lung that shares phenotypic features with B-DCs but has higher endocytic capacity. Unlike blood cells, mononuclear cells freshly isolated from lung digests exhibited markers of APCs. Furthermore, GM-CSF and IL-4 supplementation during DC cultivation induced DC differentiation and significantly increased the endocytic capacity of L-DCs. Although our L-DCs likely do not represent pure tissue resident DCs and were exposed to cytokine supplementation, we nevertheless identified an alternative way to generate airway DCs that share features with primary tissue resident DCs and may be an ideal tool for studying respiratory disease in horses.

## Abbreviations

ANOVA: Analysis of variance
APC: Antigen-presenting cells
B-DC: Blood dendritic cells
BLA 36: B lymphocyte antigen 36
B-MOS: Adherent blood monocytes
DC: Dendritic cells
EDTA: Ethylenediaminetetraacetic acid
FACS: Fluorescence-activated cell sorting
FSC: Forward scatter
GM-CSF: Granulocyte macrophage-colony stimulating factor
ICC: Immunocytochemistry
IgG: Immunoglobulin G
IL-4: Interleukin-4
L-DC: Lung dendritic cells
L-MC: Lung mononuclear cells
L-MOS: Adherent lung monocytes
mAb: Monoclonal antibodies
M-CSF: Macrophage-colony stimulating factor
MFI: Mean fluorescence intensity
MHC I & II: Major histocompatibility complex class I & II
PBMC: Peripheral blood mononuclear cells
PBS: Phosphate-buffered saline
SSC: Side scatter
TNFα: Tumor necrosis factor alpha
